# Trichodermaerin: a diterpene lactone from *Trichoderma asperellum*

**DOI:** 10.1107/S1600536814004632

**Published:** 2014-03-08

**Authors:** Suchada Chantrapromma, Chotika Jeerapong, Worrapong Phupong, Ching Kheng Quah, Hoong-Kun Fun

**Affiliations:** a Department of Chemistry, Faculty of Science, Prince of Songkla University, Hat-Yai, Songkhla 90112, Thailand; b School of Science, Walailak University, Thasala, Nakhon Si Thammarat 80160, Thailand; c X-ray Crystallography Unit, School of Physics, Universiti Sains Malaysia, 11800 USM, Penang, Malaysia

## Abstract

The title compound, C_20_H_28_O_3_, known as ‘trichodermaerin’ [systematic name: (4*E*)-4,9,15,16,16-penta­methyl-6-oxa­tetra­cyclo­[10.3.1.0^1,10^.0^5,9^]hexa­dec-4-ene-7,13-dione], is a diterpene lactone which was isolated from *Trichoderma asperellum*. The structure has a tetra­cycic 6–5–7–5 ring system, with the cyclo­hexa­none ring adopting a twisted half-chair conformation and the cyclo­pentane ring adopting a half-chair conformation, whereas the cyclo­heptene and tetra­hydro­furan­anone rings are in chair and envelope (with the methyl-substituted C atom as the flap) conformations, respectively. The three-dimensional architecture is stabilized by C—H⋯O inter­actions.

## Related literature   

For standard bond-length data, see: Allen *et al.* (1987 ▶). For ring conformations, see: Cremer & Pople (1975 ▶). For background to *Trichoderma* and diterpene lactones, see, for example: De los Santos-Villalobos *et al.* (2011 ▶); Evidente *et al.* (2006 ▶); Hajieghrari *et al.* (2008 ▶); Kumar *et al.* (2012 ▶); Vinale (2009 ▶); Xie *et al.* (2013 ▶). For the stability of the temperature controller used in the data collection, see: Cosier & Glazer (1986 ▶).
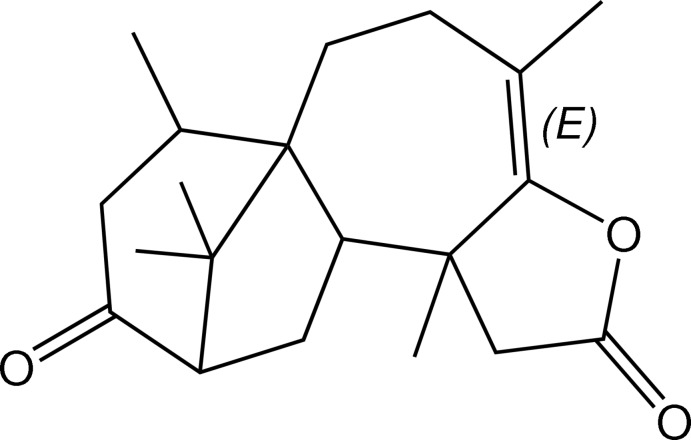


## Experimental   

### 

#### Crystal data  

C_20_H_28_O_3_
*M* *_r_* = 316.42
Monoclinic, 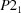

*a* = 9.1703 (4) Å
*b* = 10.2234 (5) Å
*c* = 9.2681 (4) Å
β = 108.539 (1)°
*V* = 823.81 (6) Å^3^
*Z* = 2
Mo *K*α radiation
μ = 0.08 mm^−1^
*T* = 100 K
0.47 × 0.28 × 0.17 mm

#### Data collection  

Bruker APEX DUO CCD area-detector diffractometer
Absorption correction: multi-scan (*SADABS*; Bruker, 2009 ▶) *T*_min_ = 0.962, *T*_max_ = 0.986
17364 measured reflections
2517 independent reflections
2492 reflections with *I* > 2σ(*I*)
*R*_int_ = 0.022

#### Refinement  

*R*[*F* ^2^ > 2σ(*F* ^2^)] = 0.026
*wR*(*F* ^2^) = 0.069
*S* = 1.06
2517 reflections
213 parameters
1 restraint
H-atom parameters constrained
Δρ_max_ = 0.29 e Å^−3^
Δρ_min_ = −0.16 e Å^−3^

### 

Data collection: *APEX2* (Bruker, 2009 ▶); cell refinement: *SAINT* (Bruker, 2009 ▶); data reduction: *SAINT*; program(s) used to solve structure: *SHELXTL* (Sheldrick, 2008 ▶); program(s) used to refine structure: *SHELXTL*; molecular graphics: *SHELXTL*; software used to prepare material for publication: *SHELXTL*, *PLATON* (Spek, 2009 ▶), *Mercury* (Macrae *et al.*, 2006 ▶) and *publCIF* (Westrip, 2010 ▶).

## Supplementary Material

Crystal structure: contains datablock(s) global, I. DOI: 10.1107/S1600536814004632/hb7180sup1.cif

Structure factors: contains datablock(s) I. DOI: 10.1107/S1600536814004632/hb7180Isup2.hkl

Supporting information file. DOI: 10.1107/S1600536814004632/hb7180Isup3.cml

CCDC reference: 989255

Additional supporting information:  crystallographic information; 3D view; checkCIF report

## Figures and Tables

**Table 1:** Hydrogen-bond geometry (Å, °)

*D*—H⋯*A*	*D*—H	H⋯*A*	*D*⋯*A*	*D*—H⋯*A*
C3—H3*B*⋯O3^i^	0.99	2.60	3.5649 (14)	165
C16—H16*B*⋯O2^ii^	0.98	2.41	3.2810 (16)	148
C20—H20*C*⋯O3^iii^	0.98	2.56	3.5292 (15)	173

Symmetry codes: (i) 
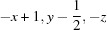
; (ii) 
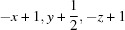
; (iii) 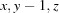.

**Fig. 1.**
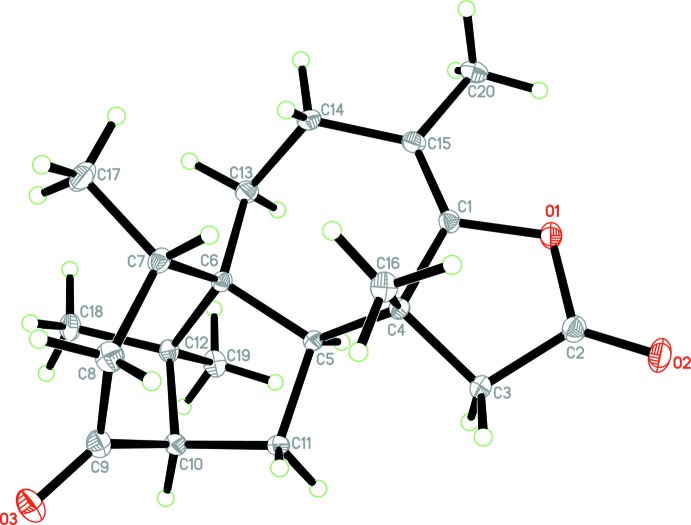
The molecular structure of the title compound, showing 40% probability displacement ellipsoids.

**Fig. 2.**
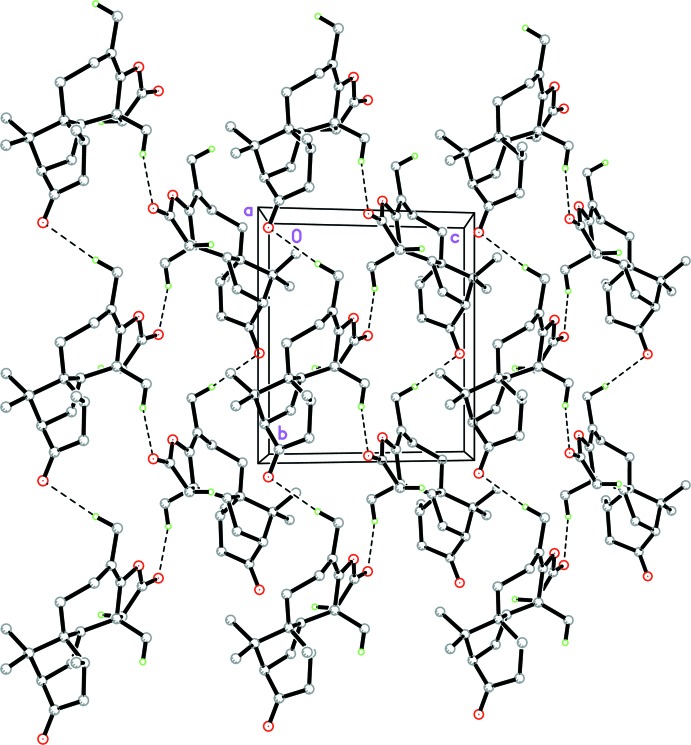
The crystal packing of the title compound viewed along the a axis, showing screw chains. Only H atoms involved in hydrogen bonds were shown for clarity. Hydrogen bonds are shown as dashed lines.

**Fig. 3.**
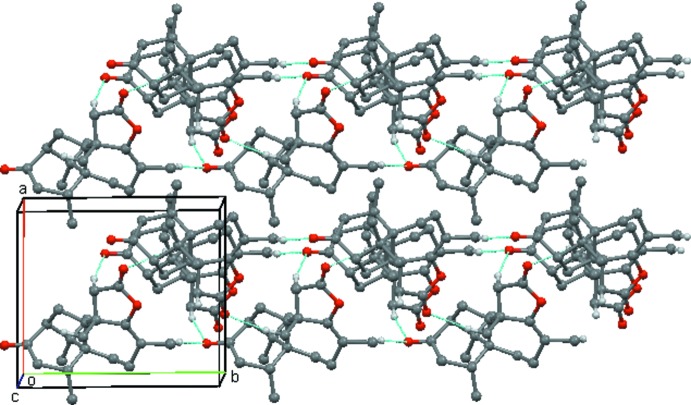
Showing the connection of the adjacent screw chains by C—H···O weak interactions. Only H atoms involved in hydrogen bonds were shown for clarity. Hydrogen bonds are shown as dashed lines.

## supplementary crystallographic information

### 1. Comment

*Trichoderma* genus is accepted as a superior biocontrol agent of plant pathogens (Hajieghrari *et al.*, 2008; Kumar *et al.*, 2012). Secondary metabolites from *Trichoderma* fungi have been reported to inhibit the phytopathogenic growth against *Colletotrichum gloeosporioides* (De los Santos-Villalobos *et al.*, 2011), *Pythium irregular*, *Sclerotinia sclerotiorum*, *Rhizoctonia solani* (Vinale, 2009) and *Sclerotium rolfsii* (Evidente *et al.*, 2006). Our study on the chemical constituents and bioactive compounds from *Trichoderma asperellum* stain F009, collected from soils in Suphan Buri province (Thailand), has led us to the isolation of the title diterpene lactone (I) which is known as "Trichodermaerin". The title compound was briefly reported together with *Trichoderma erinaceum* (Xie *et al.*, 2013). Our antifungal assay revealed that at 200 ppm of the extract had 76.5% growth inhibition against *Colletotrichum gloeosporioides*. Herein we report the crystal structure of (I).

The molecule of the title compound has a tetracyclic 6-5-7-5 ring system (Fig. 1). The cyclohexanone ring adopts a twisted half-chair conformation with the puckered C8 and C12 atoms having the maximum deviation of -0.003 (1) and 0.440 (1) Å, respectively from the best plane of the remaining four atoms (C6/C7/C9/C10) and with the puckering parameters Q = 0.6548 (12) Å, θ = 143.53 (10)° and φ = 107.15 (18)°. The cyclopentane ring is in a half-chair conformation with the puckered C6 and C12 atoms having the maximum deviation of 0.309 (1) and -0.301 (1) Å, respectively from the mean plane of C5/C10/C11 atoms and with the puckering parameters Q = 0.5030 (12) Å and φ = 52.10 (13)°. The cycloheptene ring adopts a standard chair conformation with the puckering parameter Q = 0.6792 (12) Å whereas the tetrahydrofuranone ring is in an envelope conformation with the puckered C4 atom having a deviation of 0.149 (1) Å and the puckering parameters Q = 0.2519 (12) Å and θ = 267.5 (2)° (Cremer & Pople 1975). The bond distances are of normal values (Allen *et al.*, 1987).

In the crystal structure (Fig. 2), the molecules are linked into screw chains through weak C16—H16B···O2 and C20—H20C···O3 interactions (Table 1) and the adjacent chains are further interconnected by weak C3—H3B···O3 interactions (Fig. 3 and Table 1). The crystal of (I) is consolidated by these intermolecular C—H···O weak interactions.

### 2. Experimental

*Trichoderma asperellum* stain F009 was inoculated in potato dextrose broth (PDB) for 27 days at room temperature. The broth culture (18 L) was extracted with ethyl acetate to obtain a crude ethyl acetate extract (1.956 g) as a brown viscous liquid. The crude extract was submitted to purification by column chromatography on silica gel with solvent mixtures of increasing polarity (hexane to CH_3_OH) to give fourteen fractions (F1-F14). Further separation of the subfraction F4 (122.4 mg) on silica gel column chromatography eluted with 20% ethyl acetate–hexane afforded compound (I) (5.6 mg). Colorless block-shaped single crystals of (I) suitable for *X*-ray structure determination were recrystallized from ethyl acetate by the slow evaporation of the solvent at room temperature after several days, Mp. 484.05–484.95 K.

### 3. Refinement

All H atoms were placed in calculated positions with d(C—H) = 1.00 Å for CH, 0.99 Å for CH_2_ and 0.98 Å for CH_3_ atoms. The *U*_iso_ values were constrained to be 1.5*U*_eq_ of the carrier atom for methyl H atoms and 1.2*U*_eq_ for the remaining H atoms. A rotating group model was used for the methyl groups. A total of 2218 Friedel pairs were merged before final refinement as there is no large anomalous dispersion for the determination of the absolute configuration.

### Crystal data

**Table:**

C_20_H_28_O_3_	*F*(000) = 344
*M**_r_* = 316.42	*D*_x_ = 1.276 Mg m^−^^3^
Monoclinic, *P*2_1_	Melting point = 484.05–484.95 K
Hall symbol: P 2yb	Mo *K*α radiation, λ = 0.71073 Å
*a* = 9.1703 (4) Å	Cell parameters from 2517 reflections
*b* = 10.2234 (5) Å	θ = 2.3–30.0°
*c* = 9.2681 (4) Å	µ = 0.08 mm^−^^1^
β = 108.539 (1)°	*T* = 100 K
*V* = 823.81 (6) Å^3^	Block, colorless
*Z* = 2	0.47 × 0.28 × 0.17 mm

### Data collection

**Table:**

Bruker APEX DUO CCD area-detector diffractometer	2517 independent reflections
Radiation source: sealed tube	2492 reflections with *I* > 2σ(*I*)
Graphite monochromator	*R*_int_ = 0.022
φ and ω scans	θ_max_ = 30.0°, θ_min_ = 2.3°
Absorption correction: multi-scan (*SADABS*; Bruker, 2009)	*h* = −12→12
*T*_min_ = 0.962, *T*_max_ = 0.986	*k* = −14→14
17364 measured reflections	*l* = −13→12

### Refinement

**Table:**

Refinement on *F*^2^	Primary atom site location: structure-invariant direct methods
Least-squares matrix: full	Secondary atom site location: difference Fourier map
*R*[*F*^2^ > 2σ(*F*^2^)] = 0.026	Hydrogen site location: inferred from neighbouring sites
*wR*(*F*^2^) = 0.069	H-atom parameters constrained
*S* = 1.06	*w* = 1/[σ^2^(*F*_o_^2^) + (0.0476*P*)^2^ + 0.0833*P*] where *P* = (*F*_o_^2^ + 2*F*_c_^2^)/3
2517 reflections	(Δ/σ)_max_ = 0.001
213 parameters	Δρ_max_ = 0.29 e Å^−^^3^
1 restraint	Δρ_min_ = −0.16 e Å^−^^3^

### Special details

**Table:**

Experimental. The crystal was placed in the cold stream of an Oxford Cryosystems Cobra open-flow nitrogen cryostat (Cosier & Glazer, 1986) operating at 100.0 (1) K.
Geometry. All esds (except the esd in the dihedral angle between two l.s. planes) are estimated using the full covariance matrix. The cell esds are taken into account individually in the estimation of esds in distances, angles and torsion angles; correlations between esds in cell parameters are only used when they are defined by crystal symmetry. An approximate (isotropic) treatment of cell esds is used for estimating esds involving l.s. planes.
Refinement. Refinement of F^2^ against ALL reflections. The weighted R-factor wR and goodness of fit S are based on F^2^, conventional R-factors R are based on F, with F set to zero for negative F^2^. The threshold expression of F^2^ > 2sigma(F^2^) is used only for calculating R-factors(gt) etc. and is not relevant to the choice of reflections for refinement. R-factors based on F^2^ are statistically about twice as large as those based on F, and R- factors based on ALL data will be even larger.

### Fractional atomic coordinates and isotropic or equivalent isotropic displacement parameters (Å^2^)

**Table:**

	*x*	*y*	*z*	*U*_iso_*/*U*_eq_	
O1	0.56994 (9)	0.42031 (8)	0.42963 (9)	0.01666 (16)	
O2	0.36991 (11)	0.48960 (10)	0.49811 (11)	0.0247 (2)	
O3	0.76108 (11)	1.06399 (9)	0.03459 (10)	0.02325 (19)	
C1	0.67439 (12)	0.47448 (10)	0.36106 (11)	0.01291 (18)	
C2	0.46151 (13)	0.51131 (11)	0.43292 (12)	0.01624 (19)	
C3	0.47893 (11)	0.63000 (11)	0.34415 (12)	0.01470 (19)	
H3A	0.4634	0.7112	0.3955	0.018*	
H3B	0.4043	0.6281	0.2400	0.018*	
C4	0.64611 (11)	0.62098 (10)	0.33997 (11)	0.01189 (17)	
C5	0.64570 (11)	0.66703 (10)	0.18078 (11)	0.01136 (17)	
H5A	0.5761	0.6033	0.1095	0.014*	
C6	0.79247 (11)	0.66867 (11)	0.13020 (11)	0.01212 (17)	
C7	0.92305 (11)	0.75723 (11)	0.23495 (11)	0.01437 (19)	
H7A	0.9311	0.7310	0.3412	0.017*	
C8	0.88414 (13)	0.90599 (12)	0.22599 (13)	0.0189 (2)	
H8A	0.8515	0.9288	0.3149	0.023*	
H8B	0.9798	0.9552	0.2359	0.023*	
C9	0.76116 (13)	0.95343 (12)	0.08374 (12)	0.0166 (2)	
C10	0.63987 (12)	0.85336 (11)	0.01091 (11)	0.01396 (19)	
H10A	0.5598	0.8898	−0.0805	0.017*	
C11	0.56749 (12)	0.80094 (11)	0.12984 (12)	0.01427 (19)	
H11A	0.5880	0.8615	0.2174	0.017*	
H11B	0.4549	0.7904	0.0837	0.017*	
C12	0.72306 (12)	0.73384 (11)	−0.03161 (11)	0.01303 (18)	
C13	0.84953 (12)	0.52859 (11)	0.12202 (12)	0.0158 (2)	
H13A	0.9357	0.5320	0.0793	0.019*	
H13B	0.7653	0.4781	0.0501	0.019*	
C14	0.90411 (12)	0.45392 (12)	0.27425 (13)	0.0176 (2)	
H14A	0.9707	0.3809	0.2633	0.021*	
H14B	0.9686	0.5138	0.3528	0.021*	
C15	0.77981 (11)	0.39824 (11)	0.33248 (11)	0.01390 (19)	
C16	0.75106 (12)	0.69002 (12)	0.48303 (12)	0.0160 (2)	
H16A	0.7178	0.6679	0.5706	0.024*	
H16B	0.7446	0.7849	0.4672	0.024*	
H16C	0.8575	0.6612	0.5023	0.024*	
C17	1.08590 (12)	0.73508 (14)	0.22352 (14)	0.0221 (2)	
H17A	1.1588	0.7943	0.2938	0.033*	
H17B	1.0848	0.7526	0.1192	0.033*	
H17C	1.1173	0.6443	0.2503	0.033*	
C18	0.84033 (13)	0.77677 (12)	−0.10901 (12)	0.0170 (2)	
H18A	0.7866	0.8195	−0.2060	0.026*	
H18B	0.8958	0.7000	−0.1277	0.026*	
H18C	0.9136	0.8382	−0.0428	0.026*	
C19	0.60412 (13)	0.64867 (12)	−0.14867 (12)	0.0176 (2)	
H19A	0.5600	0.6985	−0.2429	0.026*	
H19B	0.5221	0.6234	−0.1074	0.026*	
H19C	0.6546	0.5699	−0.1702	0.026*	
C20	0.78631 (13)	0.25306 (11)	0.36025 (13)	0.0175 (2)	
H20A	0.7042	0.2277	0.4012	0.026*	
H20B	0.8863	0.2300	0.4334	0.026*	
H20C	0.7727	0.2069	0.2642	0.026*	

### Atomic displacement parameters (Å^2^)

**Table:**

	*U*^11^	*U*^22^	*U*^33^	*U*^12^	*U*^13^	*U*^23^
O1	0.0190 (3)	0.0147 (4)	0.0198 (4)	0.0004 (3)	0.0111 (3)	0.0019 (3)
O2	0.0280 (4)	0.0209 (4)	0.0337 (5)	−0.0020 (4)	0.0217 (4)	0.0015 (4)
O3	0.0322 (5)	0.0150 (4)	0.0242 (4)	−0.0030 (3)	0.0112 (3)	0.0008 (3)
C1	0.0145 (4)	0.0132 (4)	0.0119 (4)	0.0001 (3)	0.0052 (3)	0.0008 (3)
C2	0.0177 (4)	0.0148 (5)	0.0181 (4)	−0.0008 (4)	0.0084 (4)	−0.0013 (4)
C3	0.0138 (4)	0.0154 (5)	0.0172 (4)	0.0013 (4)	0.0082 (3)	0.0015 (4)
C4	0.0125 (4)	0.0122 (4)	0.0123 (4)	0.0010 (3)	0.0058 (3)	0.0003 (3)
C5	0.0112 (4)	0.0117 (4)	0.0120 (4)	0.0013 (3)	0.0049 (3)	0.0006 (3)
C6	0.0116 (4)	0.0140 (4)	0.0119 (4)	0.0012 (3)	0.0053 (3)	0.0002 (3)
C7	0.0114 (4)	0.0176 (5)	0.0136 (4)	−0.0012 (4)	0.0033 (3)	−0.0001 (4)
C8	0.0185 (5)	0.0187 (5)	0.0180 (5)	−0.0035 (4)	0.0035 (4)	−0.0027 (4)
C9	0.0203 (5)	0.0154 (5)	0.0164 (4)	−0.0006 (4)	0.0088 (4)	−0.0014 (4)
C10	0.0146 (4)	0.0135 (5)	0.0141 (4)	0.0009 (3)	0.0050 (3)	0.0018 (4)
C11	0.0145 (4)	0.0137 (5)	0.0160 (4)	0.0036 (3)	0.0068 (3)	0.0028 (4)
C12	0.0143 (4)	0.0135 (4)	0.0115 (4)	−0.0010 (3)	0.0045 (3)	0.0000 (3)
C13	0.0182 (4)	0.0152 (5)	0.0168 (4)	0.0040 (4)	0.0096 (4)	0.0010 (4)
C14	0.0156 (4)	0.0182 (5)	0.0211 (5)	0.0057 (4)	0.0088 (4)	0.0051 (4)
C15	0.0149 (4)	0.0138 (4)	0.0128 (4)	0.0023 (4)	0.0041 (3)	0.0012 (4)
C16	0.0183 (4)	0.0171 (5)	0.0128 (4)	−0.0020 (4)	0.0053 (3)	−0.0029 (4)
C17	0.0122 (4)	0.0313 (6)	0.0234 (5)	−0.0006 (4)	0.0066 (4)	0.0023 (5)
C18	0.0192 (4)	0.0198 (5)	0.0144 (4)	−0.0029 (4)	0.0085 (3)	0.0001 (4)
C19	0.0197 (4)	0.0190 (5)	0.0131 (4)	−0.0043 (4)	0.0038 (3)	−0.0013 (4)
C20	0.0191 (5)	0.0131 (5)	0.0184 (5)	0.0033 (4)	0.0033 (4)	0.0005 (4)

### Geometric parameters (Å, º)

**Table:**

O1—C2	1.3691 (14)	C10—H10A	1.0000
O1—C1	1.4195 (12)	C11—H11A	0.9900
O2—C2	1.2012 (14)	C11—H11B	0.9900
O3—C9	1.2186 (15)	C12—C18	1.5343 (14)
C1—C15	1.3323 (14)	C12—C19	1.5403 (15)
C1—C4	1.5220 (15)	C13—C14	1.5410 (15)
C2—C3	1.5027 (16)	C13—H13A	0.9900
C3—C4	1.5486 (14)	C13—H13B	0.9900
C3—H3A	0.9900	C14—C15	1.5185 (15)
C3—H3B	0.9900	C14—H14A	0.9900
C4—C16	1.5398 (14)	C14—H14B	0.9900
C4—C5	1.5476 (14)	C15—C20	1.5043 (16)
C5—C11	1.5480 (14)	C16—H16A	0.9800
C5—C6	1.5597 (13)	C16—H16B	0.9800
C5—H5A	1.0000	C16—H16C	0.9800
C6—C13	1.5349 (15)	C17—H17A	0.9800
C6—C7	1.5674 (14)	C17—H17B	0.9800
C6—C12	1.5783 (14)	C17—H17C	0.9800
C7—C17	1.5470 (15)	C18—H18A	0.9800
C7—C8	1.5582 (17)	C18—H18B	0.9800
C7—H7A	1.0000	C18—H18C	0.9800
C8—C9	1.5165 (16)	C19—H19A	0.9800
C8—H8A	0.9900	C19—H19B	0.9800
C8—H8B	0.9900	C19—H19C	0.9800
C9—C10	1.5040 (15)	C20—H20A	0.9800
C10—C11	1.5517 (14)	C20—H20B	0.9800
C10—C12	1.5561 (15)	C20—H20C	0.9800
			
C2—O1—C1	110.06 (9)	C5—C11—H11B	110.7
C15—C1—O1	119.76 (10)	C10—C11—H11B	110.7
C15—C1—C4	130.91 (10)	H11A—C11—H11B	108.8
O1—C1—C4	109.25 (9)	C18—C12—C19	106.08 (8)
O2—C2—O1	120.97 (11)	C18—C12—C10	111.51 (9)
O2—C2—C3	129.62 (11)	C19—C12—C10	109.14 (8)
O1—C2—C3	109.40 (9)	C18—C12—C6	115.73 (8)
C2—C3—C4	104.12 (9)	C19—C12—C6	114.33 (9)
C2—C3—H3A	110.9	C10—C12—C6	99.96 (8)
C4—C3—H3A	110.9	C6—C13—C14	115.69 (9)
C2—C3—H3B	110.9	C6—C13—H13A	108.4
C4—C3—H3B	110.9	C14—C13—H13A	108.4
H3A—C3—H3B	109.0	C6—C13—H13B	108.4
C1—C4—C16	107.83 (9)	C14—C13—H13B	108.4
C1—C4—C5	111.74 (8)	H13A—C13—H13B	107.4
C16—C4—C5	119.53 (9)	C15—C14—C13	116.69 (9)
C1—C4—C3	100.66 (8)	C15—C14—H14A	108.1
C16—C4—C3	107.69 (8)	C13—C14—H14A	108.1
C5—C4—C3	107.66 (8)	C15—C14—H14B	108.1
C4—C5—C11	114.85 (8)	C13—C14—H14B	108.1
C4—C5—C6	123.30 (8)	H14A—C14—H14B	107.3
C11—C5—C6	105.02 (8)	C1—C15—C20	122.34 (10)
C4—C5—H5A	103.8	C1—C15—C14	121.70 (10)
C11—C5—H5A	103.8	C20—C15—C14	115.93 (9)
C6—C5—H5A	103.8	C4—C16—H16A	109.5
C13—C6—C5	110.19 (8)	C4—C16—H16B	109.5
C13—C6—C7	111.36 (8)	H16A—C16—H16B	109.5
C5—C6—C7	112.48 (8)	C4—C16—H16C	109.5
C13—C6—C12	112.81 (8)	H16A—C16—H16C	109.5
C5—C6—C12	99.45 (8)	H16B—C16—H16C	109.5
C7—C6—C12	110.03 (8)	C7—C17—H17A	109.5
C17—C7—C8	110.42 (9)	C7—C17—H17B	109.5
C17—C7—C6	115.91 (9)	H17A—C17—H17B	109.5
C8—C7—C6	114.45 (8)	C7—C17—H17C	109.5
C17—C7—H7A	104.9	H17A—C17—H17C	109.5
C8—C7—H7A	104.9	H17B—C17—H17C	109.5
C6—C7—H7A	104.9	C12—C18—H18A	109.5
C9—C8—C7	116.93 (9)	C12—C18—H18B	109.5
C9—C8—H8A	108.1	H18A—C18—H18B	109.5
C7—C8—H8A	108.1	C12—C18—H18C	109.5
C9—C8—H8B	108.1	H18A—C18—H18C	109.5
C7—C8—H8B	108.1	H18B—C18—H18C	109.5
H8A—C8—H8B	107.3	C12—C19—H19A	109.5
O3—C9—C10	123.42 (10)	C12—C19—H19B	109.5
O3—C9—C8	122.25 (11)	H19A—C19—H19B	109.5
C10—C9—C8	114.33 (10)	C12—C19—H19C	109.5
C9—C10—C11	109.78 (9)	H19A—C19—H19C	109.5
C9—C10—C12	107.20 (8)	H19B—C19—H19C	109.5
C11—C10—C12	105.28 (8)	C15—C20—H20A	109.5
C9—C10—H10A	111.4	C15—C20—H20B	109.5
C11—C10—H10A	111.4	H20A—C20—H20B	109.5
C12—C10—H10A	111.4	C15—C20—H20C	109.5
C5—C11—C10	105.11 (8)	H20A—C20—H20C	109.5
C5—C11—H11A	110.7	H20B—C20—H20C	109.5
C10—C11—H11A	110.7		
			
C2—O1—C1—C15	−173.20 (10)	C7—C8—C9—O3	−150.53 (11)
C2—O1—C1—C4	9.64 (11)	C7—C8—C9—C10	30.16 (14)
C1—O1—C2—O2	−173.44 (10)	O3—C9—C10—C11	−124.95 (11)
C1—O1—C2—C3	7.53 (12)	C8—C9—C10—C11	54.36 (12)
O2—C2—C3—C4	160.06 (12)	O3—C9—C10—C12	121.19 (12)
O1—C2—C3—C4	−21.02 (11)	C8—C9—C10—C12	−59.51 (11)
C15—C1—C4—C16	−85.57 (13)	C4—C5—C11—C10	156.08 (8)
O1—C1—C4—C16	91.17 (9)	C6—C5—C11—C10	17.42 (10)
C15—C1—C4—C5	47.72 (15)	C9—C10—C11—C5	−100.89 (10)
O1—C1—C4—C5	−135.54 (8)	C12—C10—C11—C5	14.19 (10)
C15—C1—C4—C3	161.77 (11)	C9—C10—C12—C18	−45.64 (11)
O1—C1—C4—C3	−21.50 (10)	C11—C10—C12—C18	−162.50 (8)
C2—C3—C4—C1	24.59 (10)	C9—C10—C12—C19	−162.51 (9)
C2—C3—C4—C16	−88.18 (10)	C11—C10—C12—C19	80.63 (10)
C2—C3—C4—C5	141.69 (9)	C9—C10—C12—C6	77.25 (9)
C1—C4—C5—C11	161.08 (8)	C11—C10—C12—C6	−39.61 (9)
C16—C4—C5—C11	−71.71 (12)	C13—C6—C12—C18	−74.27 (11)
C3—C4—C5—C11	51.45 (11)	C5—C6—C12—C18	169.02 (9)
C1—C4—C5—C6	−68.68 (12)	C7—C6—C12—C18	50.76 (12)
C16—C4—C5—C6	58.53 (13)	C13—C6—C12—C19	49.47 (11)
C3—C4—C5—C6	−178.31 (9)	C5—C6—C12—C19	−67.25 (10)
C4—C5—C6—C13	65.63 (12)	C7—C6—C12—C19	174.50 (8)
C11—C5—C6—C13	−160.19 (8)	C13—C6—C12—C10	165.87 (8)
C4—C5—C6—C7	−59.28 (13)	C5—C6—C12—C10	49.15 (9)
C11—C5—C6—C7	74.90 (10)	C7—C6—C12—C10	−69.10 (9)
C4—C5—C6—C12	−175.68 (9)	C5—C6—C13—C14	−64.06 (11)
C11—C5—C6—C12	−41.51 (9)	C7—C6—C13—C14	61.49 (11)
C13—C6—C7—C17	39.01 (12)	C12—C6—C13—C14	−174.21 (9)
C5—C6—C7—C17	163.27 (9)	C6—C13—C14—C15	77.90 (13)
C12—C6—C7—C17	−86.84 (11)	O1—C1—C15—C20	4.36 (15)
C13—C6—C7—C8	169.33 (9)	C4—C1—C15—C20	−179.19 (10)
C5—C6—C7—C8	−66.41 (11)	O1—C1—C15—C14	−173.71 (9)
C12—C6—C7—C8	43.47 (11)	C4—C1—C15—C14	2.75 (17)
C17—C7—C8—C9	110.78 (10)	C13—C14—C15—C1	−59.24 (14)
C6—C7—C8—C9	−22.18 (13)	C13—C14—C15—C20	122.58 (11)

### Hydrogen-bond geometry (Å, º)

**Table:**

*D*—H···*A*	*D*—H	H···*A*	*D*···*A*	*D*—H···*A*
C3—H3*B*···O3^i^	0.99	2.60	3.5649 (14)	165
C16—H16*B*···O2^ii^	0.98	2.41	3.2810 (16)	148
C20—H20*C*···O3^iii^	0.98	2.56	3.5292 (15)	173

Symmetry codes: (i) −*x*+1, *y*−1/2, −*z*; (ii) −*x*+1, *y*+1/2, −*z*+1; (iii) *x*, *y*−1, *z*.
